# Supplementary material for: MetaRibo-Seq measures translation in microbiomes
Source: Nat Commun. 2020 Jun 29;11:3268. doi: 10.1038/s41467-020-17081-z (PMC7324362; doi:10.1038/s41467-020-17081-z)
Supplement: Supplementary file 10 — Supplementary Data 7 [file 41467_2020_17081_MOESM10_ESM.zip › File2/Confidence_High_Taxonomy/261518_out.krona.html]

Javascript must be enabled to view this page.

members
magnitude
magnitudeUnassigned
count
unassigned
taxon
rank

261518\_out

18

2
superkingdom
18

1239
phylum
18

186801
class
18

186802
18
order

186806
family
18

1
18
1730

SRS052697\_contig\_number\_contig-100\_1437.209078
genus

39485

SRS013940\_contig\_number\_10118SRS019693\_contig\_number\_contig-100\_2116.44541SRS024492\_contig\_number\_11640SRS075773\_contig\_number\_31758SRS077231\_contig\_number\_contig-100\_1120.82600SRS077641\_contig\_number\_9004SRS143342\_contig\_number\_1240SRS148196\_contig\_number\_42576
species
8

142586

SRS018836\_contig\_number\_25860SRS020233\_contig\_number\_53264SRS043001\_contig\_number\_contig-100\_117.68794SRS047044\_contig\_number\_17976SRS049959\_contig\_number\_42987SRS049995\_contig\_number\_contig-100\_5106.173165SRS1055049\_contig\_number\_3341SRS140492\_contig\_number\_contig-100\_1013.207187SRS147614\_contig\_number\_contig-100\_4895.4896
species
9
